# *Coxiella burnetii* Lipopolysaccharide: What Do We Know?

**DOI:** 10.3390/ijms18122509

**Published:** 2017-11-23

**Authors:** Prasad Abnave, Xavier Muracciole, Eric Ghigo

**Affiliations:** 1 Department of Zoology, University of Oxford, Tinbergen Building, South Parks Road, Oxford OX1 3PS, UK; prasadabnave@gmail.com; 2 Department of Radiotherapy Oncology, CHU de la Timone, Assistance Publique-Hopitaux Marseille, 13385 Marseille, France; xavier.muracciole@gmail.com; 3 Unité de Recherche sur les Maladies Infectieuses et Tropicales Emergentes (URMITE), Institut Hospitalier Universitaire Méditerranée-Infection, 19-21 Bd Jean Moulin, CEDEX 05, 13385 Marseille, France

**Keywords:** lipopolysaccharide, *Coxiella burnetii*, Q fever, phagosome, virenose

## Abstract

A small gram-negative bacterium, *Coxiella burnetii* (*C. burnetii*), is responsible for a zoonosis called Q fever. *C. burnetii* is an intracellular bacterium that can survive inside microbicidal cells like monocytes and macrophages by hijacking several functions of the immune system. Among several virulence factors, the lipopolysaccharide (LPS) of *C. burnetii* is one of the major factors involved in this immune hijacking because of its atypical composition and structure. Thus, the aim of this mini-review is to summarize the repressive effects of *C. burnetii* LPS on the antibacterial immunity of cells.

## 1. Introduction

*Coxiella burnetii* is an intracellular bacterium responsible for a worldwide zoonosis known as Q fever [1,2]. After primary infection, approximately 60% of humans remain asymptomatic, while 40% manifest clinical signs consisting of isolated fever, hepatitis, and pneumonia [3]. The principal clinical manifestation of Q fever is endocarditis with a lethal prognosis without treatment. The treatment involves a combination of doxycycline and hydroxychloroquine [1,3]. However, this long-term treatment carries the persistent risk of relapse [4].

*C. burnetii* is a small bacterium measuring approximately 0.2 to 0.4 µm wide and 0.4 to 1 µm long, and it has been classified in the *Proteobacteria* subdivision based on its 16S ribosomal RNA sequence. As *C. burnetii* harbours lipopolysaccharide (LPS) in its membrane, it is defined as a gram-negative bacterium. Though *C. burnetii* is not stained by Gram stain, it can be stained by Gimenez stain [5]. *C. burnetii* primarily infects domestic ruminants and pets, but arthropods have also been found to be infected. In animals, the infection is asymptomatic but induces abortions in livestock. Both abortion and parturition contribute to the bacteria spreading into the environment, since the placenta of infected animals contains large amounts of *C. burnetii*. Contamination via aerosols also remains the major route of infection in both animals and humans [3,6]. *C. burnetii* has been categorized as a biological weapon due to its high infectivity, the possibility of producing large quantities of bacteria, its environmental stability through a sporulation-like mechanism, and its dispersion via aerosolization [7,8]. *C. burnetii* was likely used as a bio-weapon during World War II, as a Q fever outbreak was observed during this time among army troops [9].

*C. burnetii* resides primarily within myeloid cells (monocytes and macrophages) [10,11] but has also been shown to infect trophoblasts [12] and adipocytes [13]. The adaptation of *C. burnetii* to its environment is probably critical for its survival. To survive within its host, *C. burnetii* interferes with the host’s antimicrobial response (immunity and phagolysosome biogenesis). For this purpose, *C. burnetii* has an arsenal of virulence factors [14,15,16,17], including LPS [18]. The molecular variations observed in *C. burnetii* LPS, a major component of its outer membrane, contribute to its pathogenic properties [19,20,21]. Moreover, the intracellular fate of virulent *C. burnetii* in myeloid cells is also determined by its LPS composition [18].

## 2. *Coxiella burnetii* LPS: Structure and Composition

As observed in enterobacteria, *C. burnetii* displays antigenic variations, from a smooth-rough form called Phase I to a rough form known as Phase II. The Phase I form is isolated from natural sources and defined as a virulent form of *C. burnetii*. It is characterized by full-length LPS and survives inside monocytes and macrophages [10,11]. After several passages of the virulent *C. burnetii* in embryonated eggs or tissue culture, an irreversible modification is observed in the molecular weight of *C. burnetii* LPS. *C. burnetii* harbouring a truncated LPS is defined as an avirulent microorganism and eliminated by monocytes and macrophages [22,23]. This avirulent form does not exist in the natural environment. It was shown that this LPS modification occurs due to a genomic deletion [3]. The difference between the virulent and avirulent forms of *C. burnetii* lies in the *O*-antigen; specifically, LPS from virulent *C. burnetii* has an *O*-antigen that contains unusual sugars, l-virenose, dihydrohydroxystreptose, and galactosamine uronyl-α-(1,6)-glucosamine residues, whereas LPS from the avirulent form does not have any *O*-antigen [19,20,21,24,25,26,27,28,29,30,31,32]. Virenose and dihydrohydroxystreptose have not been found in any other enterobacterial LPSs and are thus unique biomarkers of virulent *C. burnetii*. Interestingly, the lipid A molecules of both virulent and avirulent *C. burnetii* display the same ionic species and fragmentation profiles in mass spectrometry, suggesting that they have very similar and likely identical structures. The *C. burnetii* lipid A structure differs considerably from the published standard form of enterobacterial lipid A. An analysis of lipid A from *C. burnetii* identified two major tetra-acylated molecular species sharing the classical backbone of a dephosphorylated GlcN (acylated d-glucosamine residues) disaccharide in which both GlcN I and GlcN II carry an amide-linked iso or normal (n) C16:0(3-OH) [24,33]. The core polysaccharide is conserved between virulent and avirulent *C. burnetii* LPSs and contains a heptasaccharide localized in the proximal region of lipid A. The heptasaccharide is formed by two terminal d-mannoses (Man), 2- and 3,4-linked d-*glycero*-d-*manno*-heptoses, and terminal 4- and 4,5-linked 3-deoxy-d-*manno*-oct-2-ulosonic acid residues [20,29]. It is important to note that a third *C. burnetii* LPS has been identified as an intermediate-length LPS at the surface of the Nine Mile Crazy strain [34]. Large chromosomal deletions have been found in these avirulent *C. burnetii* Nine Mile and Nine Mile Crazy strains [35]. These deletions eliminate open reading frames involved in the biosynthesis of *O*-antigen sugars, including the rare sugar virenose [35]. The description of the virenose biosynthesis pathway suggests the formation of GDP-β-d-virenose via the modification of GDP-l-fucose by the addition of a methyl group at position C3”, and perhaps the open reading frame CBU0691, and the inversion of the stereochemistry at position C2” [36].

## 3. *C. burnetii* LPS Interferes with Phagocytosis

It is known that phagocytosis efficiency depends on the activation of phagocytic receptor CR3 (complement receptor-3) through αvβ3 integrin and CD47 (integrin-associated protein). *C. burnetii*, via its LPS, subverts receptor-mediated phagocytosis [22] by inhibiting the interplay between integrins, including CR3, remodelling the actin cytoskeleton organization, and activating protein tyrosine kinases. This strategy possibly determines the evolution of Q fever. *C. burnetii*, via its LPS, interacts with macrophages through αvβ3 integrins, and avoids internalization by inhibiting the interaction between αvβ3 integrins and CR3, which is essential for bacterial uptake [22,37]. Inhibition of the interplay between αvβ3 integrins and CR3 leads to poor internalization of virulent *C. burnetii* compared with its avirulent form, which harbours a truncated LPS and is largely internalized by monocytes and macrophages. Interestingly, the inhibitory mechanism mediated by virulent *C. burnetii* through its LPS does not target CD47 [22]. Note that CR3, not αvβ3 integrin, is excluded from the cytoskeleton protrusions formed during the cytoskeleton reorganization induced by virulent *C. burnetii* LPS*,* thus decreasing the efficiency of phagocytosis [22,37,38]. An in-depth analysis has demonstrated that the uptake of avirulent *C. burnetii* requires both CD11b/CD18 and CR3, whereas virulent organism internalization does not involve CR3. It has been shown that the LPS from virulent *C. burnetii* prevents the activation of CR3 by interfering with its lectin sites [22]. This leads to conformational changes in the I domain and in the exposure of activation epitopes and cytoskeleton reorganization [39].

Finally, virulent *C. burnetii* induces early protein tyrosine kinase activation as well as the tyrosine phosphorylation of two Src-related kinases: Hck and Lyn [40]. By contrast, the avirulent form does not stimulate protein tyrosine kinases. Tyrosine-phosphorylated proteins co-localize with F-actin inside protrusions. Cell membrane protrusions are induced via the activation of protein tyrosine kinases by *C. burnetii* LPS*,* which in turn down-modulates *C. burnetii* uptake [40,41]. The use of protein tyrosine kinase inhibitors rescues *C. burnetii* phagocytosis. It has been hypothesized that the membrane ruffling induced by protein tyrosine kinase activation may interfere with the co-localization of CR3 with αvβ3 integrin and *C. burnetii* [42,43]. It has also been shown that *C. burnetii* LPS interferes with Toll Like Receptor (TLR)-2 and TLR-4 signalling through cytoskeleton reorganization [38,41,42]. Indeed, cytoskeleton reorganization induces a redistribution of TLR-2 and TLR-4 on the membrane of macrophages. This redistribution disrupts the colocalization between TLR-2 and TLR-4, in contrast to what is observed in macrophages challenged with LPS from the avirulent strain of *C. burnetii*. Co-immunoprecipitation experiments have revealed that a possible physical link between TLR-2 and TLR-4 is broken in cells challenged with virulent *C. burnetii* LPS. As a consequence, p38α Mitogen-Activated Protein Kinase (MAPK) is not activated in macrophages challenged with virulent *C. burnetii* and LPS extracted from virulent *C. burnetii* [18,41,44]*.* However, the existence of a *TLR2/TLR4/*p38α MAPK axis in *C. burnetii* infection remains to be demonstrated.

## 4. *C. burnetii* LPS Interferes with the Antibacterial Immune Response

Macrophage immune polarization is reoriented by *C. burnetii* to deactivate the macrophage microbicidal response [45,46]. Indeed, *C. burnetii* is responsible for atypical M2 macrophage activation, and it has been shown to induce expression of M2 polarization-related genes (transforming growth factor-β1, interleukin (IL)-1 receptor antagonist, Chemokine (C-C motif) ligand (CCL)18, mannose receptor, arginase-1). By contrast, the expression of genes related to M1 polarization (tumor necrosis factor, CD80, C-C chemokine receptor type (CCR)7) is inhibited. It is interesting to note that the expression of arginase-1 is associated with the absence of nitric oxide production, while the expression of the *Interleukin (IL)-6* and *Chemokine (C-X-C motif) ligand (CXCL)8* genes (M1-related genes) is increased, although their proteins are weakly secreted [45]. In addition, monocytes produce high levels of IL-10 in response to *C. burnetii* or its LPS. IL-10 favours the persistence of *C. burnetii* by down-regulating the expression of tumor necrosis factor [47,48,49]. It is also responsible for the expression of *Programmed cell Death protein (PD)-1* by monocytes in vitro, and most likely, in patients with Q fever endocarditis. The LPS of *C. burnetii* does not induce the expression of PD-1 by monocytes. PD-1 delivers an inhibitory signal to T cells [50,51], and its expression in Q fever contributes to the immune suppression observed in Q fever endocarditis [52].

## 5. *C. burnetii* LPS as a Determinant Factor in Phagolysosome Biogenesis

In human macrophages, it has been observed that, in contrast to virulent *C. burnetii*, the avirulent form is quickly eliminated in degradative phagolysosome-like compartments [11,47]. Their replication is partially controlled in resident mouse peritoneal macrophages [53]. Immediately after phagocytosis, both virulent and avirulent forms of *C. burnetii* are localized within an early phagosome, transiently harbouring EEA1 (early endosome auto-antigen-1). This early phagosome undergoes a maturation process and is transformed into a late phagosome, presenting the markers Lamp-1, CD63, mannose-6-phosphate receptor, and V-H+ATPase and possessing an acidic pH. The major difference between the compartments containing virulent and avirulent forms of *C. burnetii* is the absence of the small GTPase Rab7 at the surface of the phagosome containing the virulent *C. burnetii* [11,17,18,23]. In contrast to the vacuole with avirulent bacteria, the phagosome containing the virulent strain of *C. burnetii* does not mature in phagolysosomes [23]. Surprisingly, the intracellular trafficking of *C. burnetii* LPS is similar to the trafficking of intact bacteria. Indeed, the LPSs from virulent and avirulent *C. burnetii* traffic through early phagosomes characterized by the presence of the small GTPase Rab5 and EEA1 [18,54]. Nevertheless, endosomes containing LPS purified from avirulent bacteria develop into late endosomes (Rab7, Lamp1) and then into lysosomes containing the lysosomal enzyme cathepsin D. The endosomes transporting LPS isolated from virulent bacteria mature from early to late endosomes but do not become lysosomes. Interestingly, in terms of intact *C. burnetii*, late endosomes containing LPS do not express the Rab7 protein on their surface [18,23,55]. This result suggests that LPS is responsible for blocking phagolysosome maturation induced by *C. burnetii*. Investigations of *C. burnetii* LPS have demonstrated that the LPS from pathogenic *C. burnetii* does not induce the phosphorylation of p38α MAPK by Mitogen-Activated Protein Kinase Kinase (MKK)6. This defect in the activation of p38α MAPK prevents the serine phosphorylation (S796E) of Vps41. In the absence of phosphorylation, Vps41 does not promote the targeting of the HOPS (homotypic fusion and protein sorting) complex to endosome–vacuole fusion sites, and thus it fails to recruit the GTP-bound Rab7 required for phagosome–lysosome fusion [56,57,58,59,60,61]. The absence of p38α MAPK activation is most likely due to the engagement of TLR4 by two unusual sugars, virenose and dihydrohydroxystreptose, present in the LPS of pathogenic *C. burnetii*. Thus, LPS from virulent *C. burnetii* acts as an antagonist of TLR-4.

## 6. Concluding Remarks

Collectively, this evidence highlights the importance of LPS and its composition in the strategies used by *C. burnetii* to infect cells and develop an efficient infection that leads to Q fever. It is interesting to observe that the particular composition of *C. burnetii* LPS allows several axes of the immune response to be modulated, ranging from phagocytosis to vesicular trafficking. Certainly, the virulence of *C. burnetii* does not only depend on LPS, as other virulence factors have been identified in *C. burnetii* [11,14]. The recent successful culturing of *C. burnetii* in axenic conditions might significantly develop our understanding of *C. burnetii* infection by facilitating the identification of new virulence factors [62,63]. Further work is required to understand the mechanisms implied in anti-microbicidal response hijacking. It might be interesting to generate transgenic *Escherichia coli* expressing the LPSs from both the virulent and avirulent *C. burnetii* to better understand LPS action. Similarly, as several new *C. burnetii* strains that cause severe Q fever have been isolated [2], it will be interesting to analyse their LPS composition to determine if the virulence and clinical issues observed are linked to any particular structure or composition of LPS.
